# Anesthetic Challenges in Debulking Surgery of Massive Plexiform Neurofibroma With Neurofibromatosis Type 1: A Case Report of Intraoperative Generalized Involuntary Movements and Severe Blood Loss

**DOI:** 10.7759/cureus.106054

**Published:** 2026-03-29

**Authors:** Ana Nido-Cebollero, Gerardo Garcia-Rivera, Harold L Rivera-Alvarado, Felix A Rullan-Lopez de Victoria, Andrés B Sosa-González, Hector Torres, Maria J Crespo

**Affiliations:** 1 Anesthesiology, University of Puerto Rico School of Medicine, San Juan, PRI; 2 Physiology, University of Puerto Rico School of Medicine, San Juan, PRI

**Keywords:** general anesthesia, intraoperative hemorrhage, neurofibromatosis 1, seizure-like episode, skin neurofibroma

## Abstract

Neurofibromatosis type 1 is a multisystem genetic disorder commonly associated with plexiform neurofibromas, benign but highly vascular tumors that present significant perioperative challenges. Surgical debulking of plexiform neurofibromas is often associated with difficult anesthetic management due to tumor vascularity, hemorrhage risk, airway considerations, and the need for intraoperative neurophysiological monitoring. We report the case of a 48-year-old man with neurofibromatosis type 1 who underwent debulking of a large right thigh plexiform neurofibroma under general anesthesia without neuromuscular blockade. The intraoperative course was notable for significant blood loss requiring transfusion and the development of transient generalized involuntary movements temporally associated with deep tumor manipulation. Despite these challenges, the procedure was successfully completed. This case highlights the anesthetic complexity of massive plexiform neurofibroma resection and underscores the importance of maintaining a broad differential diagnosis for intraoperative seizure-like phenomena in patients without epilepsy or intracranial pathology. Meticulous planning, multidisciplinary communication, and individualized anesthetic strategies are essential to optimize safety in complex neurofibromatosis type 1-related surgical procedures.

## Introduction

Neurofibromatosis type 1 (NF1) is an autosomal dominant familial tumor syndrome characterized by abnormalities in skin pigmentation and the development of tumors along peripheral nerves in the skin, brain, and other organs [1-2]. This syndrome results from loss-of-function mutations in the *NF1 *gene, which encodes neurofibromin, leading to dysregulated cellular growth and tumor formation [3]. The condition has an estimated incidence of approximately one in 3,000 individuals and a prevalence of one in 2,000, reflecting the increased mortality associated with the disease [4]. Clinical expressions are highly variable, ranging from mild cutaneous involvement to severe, multisystem disease.

Neurofibromatosis type 1 is diagnosed when two or more of the following characteristic features are present: six or more café-au-lait macules measuring >5 mm in diameter in prepubertal individuals or >15 mm in postpubertal individuals, axillary or inguinal freckling, two or more neurofibromas of any type or one plexiform neurofibroma (PN), two or more Lisch nodules of the iris, a distinctive osseous lesion, an optic pathway glioma, or a first-degree relative with NF1 diagnosed according to these criteria [5].

Plexiform neurofibromas are peripheral nerve sheath tumors that are typically congenital and may continue to grow throughout adolescence and early adulthood [6-7]. Among individuals with NF1, approximately one-third develop clinically visible plexiform neurofibromas [8], while imaging studies suggest that up to half of patients may harbor internal tumors detectable only radiographically [9]. These tumors may infiltrate surrounding tissues, leading to substantial pain, bone destruction, disfigurement, neurological deficits, and impaired quality of life [6-7]. PNs carry an estimated lifetime risk of malignant transformation into malignant peripheral nerve sheath tumors of 15.8% [10]. Surgical resection remains the primary therapeutic option for symptomatic lesions; however, complete excision is often not feasible, and chemotherapy has been utilized in selected cases [7]. Early surgical intervention is recommended, as neurofibromas tend to grow more rapidly in younger patients, and earlier excision may reduce the risk of malignant transformation [11]. Nonetheless, complete resection is rarely achieved, and tumor regrowth rates remain high [12].

From an anesthetic perspective, patients with NF1 present significant perioperative challenges due to the disorder’s multisystem involvement. Airway, cardiovascular, respiratory, neurological, and gastrointestinal abnormalities are common and may complicate anesthetic evaluation and intraoperative management, necessitating individualized perioperative planning [13]. In addition to these patient-related considerations, PNs pose distinct surgical and anesthetic challenges. Owing to their highly vascular nature, resection is frequently complicated by substantial intraoperative blood loss and hemorrhagic events [14]. Accordingly, careful preoperative planning and heightened intraoperative preparedness for complications are essential.

In this context, this case is presented to highlight the anesthetic complexity associated with the resection of massive PNs in patients with NF1, particularly when intraoperative neurophysiological monitoring is employed. It emphasizes the occurrence of atypical intraoperative neuromuscular phenomena during tumor manipulation in the absence of corresponding electrodiagnostic abnormalities, an observation that remains insufficiently characterized in the literature. By reporting this case, we aim to expand current understanding of perioperative challenges in NF1 and to increase awareness of uncommon intraoperative findings that may influence anesthetic management and clinical decision-making.

## Case presentation

A 48-year-old male with a medical history of NF1 and hypothyroidism presented in April 2025 to the surgical facilities of the Puerto Rico Medical Center for elective debulking of a recurrent right thigh PN extending along the distribution of the lateral femoral cutaneous nerve.

His surgical history was notable for two prior extensive PN resections at the same anatomical location, performed 20 and 17 years before the current procedure. Family history was remarkable for a brother with NF1, with clinical manifestations confined to the right forearm. Neither the patient nor his brother had a known history of surgical complications, adverse reactions to anesthesia, seizures, or involuntary movements. The patient denied any allergies, and his home medication included levothyroxine 50 mcg orally once daily.

**Figure 1:**
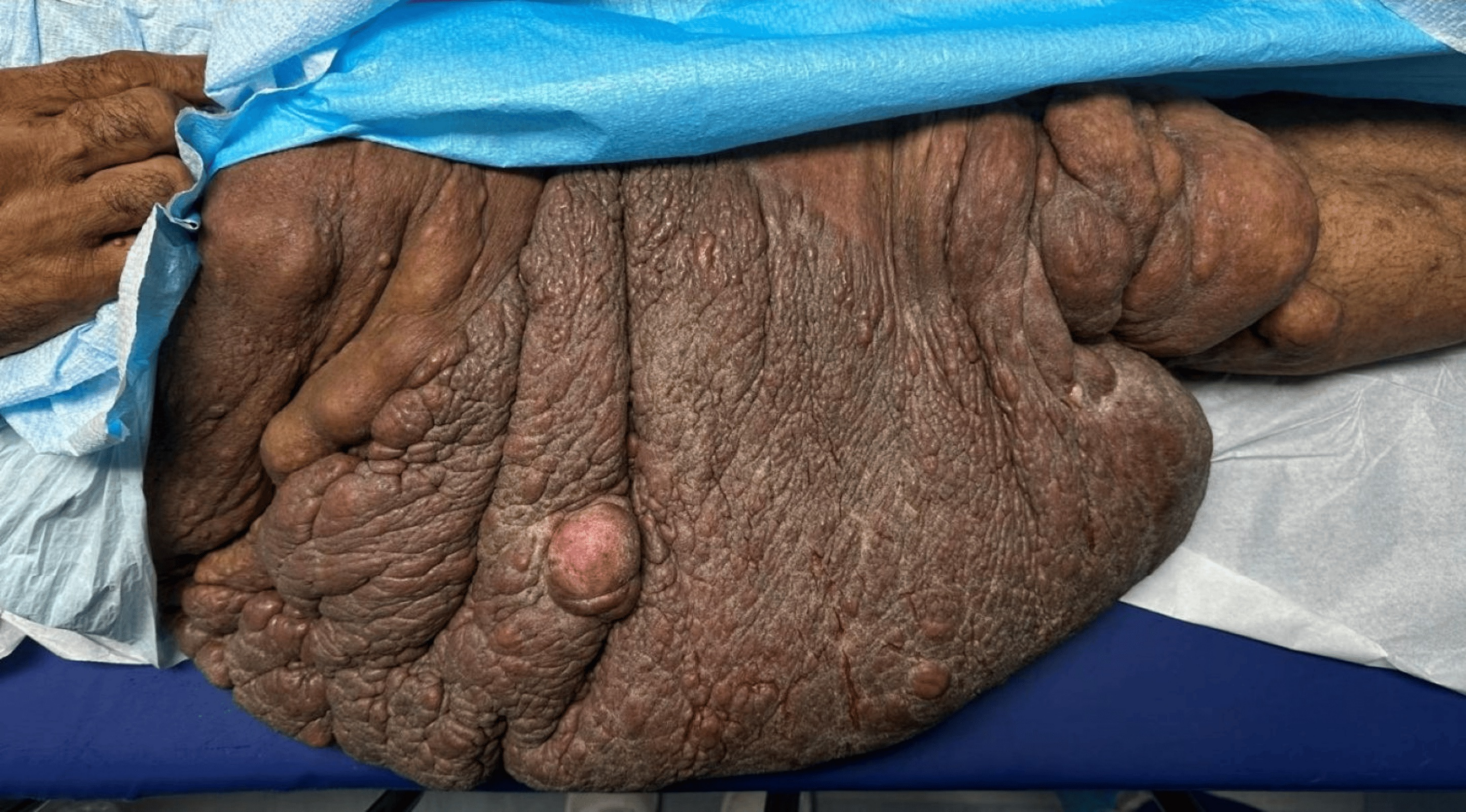
Pre-operative image of plexiform neurofibroma.

During the preoperative evaluation, he was alert, oriented, and fasted for over 8 hours. The patient weighed 99.79 kg and was 185.42 cm tall. Vital signs included a heart rate (HR) of 62 bpm, blood pressure (BP) of 140/79 mmHg, and peripheral oxygen saturation (SpO2) 100% on room air. Patient referred diminished sensation on the right thigh, and shortness of breath upon exertion secondary to the neurofibroma, with two episodes of loss of consciousness. Physical exam was remarkable for a massive PN involving the right anteromedial thigh extending from the inguinal canal to the upper border of the knee, measuring approximately 62 x 35 cm (Figure 1).

There were many small neurofibromas throughout his body. Airway examination revealed a Mallampati class III, no visible tumor growths, and normal mouth opening and neck mobility [15]. He denied previous history of difficult airway. Preoperative laboratory studies (Table 1) were unremarkable. Electrocardiogram and chest radiograph findings were within normal limits. The patient was classified as American Society of Anesthesiologists (ASA) physical status II [16].

**Table 1:** Preoperative Laboratory Results SGPT: Serum glutamic-pyruvic transaminase, SGOT: Serum glutamic-oxaloacetic transaminase, AST: Aspartate transaminase, ALT: Alanine transaminase.

Test	Results	Normal Range
White Blood Count (X 10^3/uL)	8.34	3.98 - 10.04
Red Blood Count (X 10^6/uL)	4.47	3.93 - 5.22
Hemoglobin (gm/dL)	12.9	11.2 - 15.1
Hematocrit (%)	40	36 - 44
Platelets (X 10^3/uL)	292	163- 369
Prothrombin Time (seconds)	13.1	9.00 - 11.50
Partial Thromboplastin Time (seconds)	27	22.20 - 34.00
International Normalized Ratio (INR)	0.9	0.90 - 1.10
Sodium (mEq/L)	139	135 - 145
Potassium (mEq/L)	4.9	3.30 - 5.10
Chloride (mEq/L)	103	98.00 - 107.00
Carbon Dioxide, Total (mEq/L)	27	25.00 - 30.00
Blood Urea Nitrogen (mg/dL)	15	7.00 - 20.00
Creatinine (mg/dL)	0.9	0.50 - 1.50
Glucose (mg/dL)	99	70.00 - 99.00
Calcium (mg/dL)	9.2	8.6 - 10.2
Alkaline Phosphatase (u/L)	57	38.00 - 126.00
SGPT (ALT) (u/L)	9	0.0 - 35.00
SGOT (AST) (u/L)	9	15.00 - 46.00
Albumin (g/dL)	4.1	3.50 - 5.00

The patient was premedicated with intravenous (IV) midazolam (2.5 mg) for anxiolysis and was transported to the operating room. The patient was moved to the operating bed in the supine position. Standard ASA monitors were applied. Following adequate preoxygenation, induction was achieved through a 20-gauge peripheral IV catheter in the left arm using fentanyl (50 mcg, IV), propofol (200 mg, IV), and succinylcholine (100 mg, IV). Although succinylcholine was administered during anesthetic induction, additional neuromuscular blockade was avoided to allow reliable intraoperative neurophysiological monitoring (IONM), including somatosensory evoked potentials (SSEPs) and motor evoked potentials (MEPs) of the relevant lower extremity muscle groups. The patient was then mask ventilated and intubated using a McGRATH™ MAC4 video laryngoscope (Medtronic, Minneapolis, USA) with a 7.5 mm endotracheal tube. Intubation was accomplished with moderate difficulty, corresponding to a Cormack-Lehane grade IIa view [15]. Induction and subsequent ventilation were uneventful. Mechanical ventilation was initiated with a tidal volume of 550 mL and a respiratory rate of 15 breaths per minute. Anesthesia was maintained with sevoflurane at 2% in a 50% oxygen/50% air mixture, with a minimum alveolar concentration (MAC) of 0.4 throughout the procedure and a total fresh gas flow of 2 L/min. Invasive arterial monitoring was established via a left radial arterial line. Additional vascular access included three peripheral intravenous catheters: 18-gauge in the right arm, a 20-gauge in the right hand, and a 16-gauge catheter in the right external jugular vein, for a total of four venous access points. Anesthesia was maintained with continuous IV administration of propofol (100 mcg/kg/min), ketamine (10 mcg/kg/min), and lidocaine (4 mg/min). In addition, famotidine (20 mg, IV), cefazolin (2 g, IV), dexamethasone (4 mg, IV), tranexamic acid (TXA, 1 g, IV), and glycopyrrolate (0.4 mg, IV) were administered. Fentanyl boluses (50 mcg, IV) were given during the procedure for pain management.

Fifty-five minutes into the procedure, the patient developed generalized involuntary jerking movements that corresponded with surgical manipulation of structures deep within the neurofibroma adjacent to the femoral artery. The movements consisted of brief, twitch-like bursts of shaking and muscular contractions lasting from less than one to several seconds. They involved different body regions across episodes and occurred sporadically without a discernible pattern. Although noticeable to the surgical team, their intensity remained limited and did not displace the patient or compromise positioning on the operating table. No abnormalities were observed by IONM during these episodes. Multiple interventions were attempted to suppress the movements, including increasing the propofol infusion to 200 mcg/kg/min, titrating the ketamine infusion to 30 mcg/kg/min, and initiating an IV fentanyl infusion at 1 mcg/kg/hr, later increased to 5 mcg/kg/hr. Despite these adjustments, the involuntary movements persisted for approximately 30 minutes and ended spontaneously. During the episodes, vital signs remained stable. Following cessation of the movements, the propofol infusion was reduced to 125 mcg/kg/min, ketamine to 10 mcg/kg/min, and fentanyl to 1 mcg/kg/hr. Sevoflurane was maintained at a MAC of 0.4 throughout the entire procedure.

**Figure 2:**
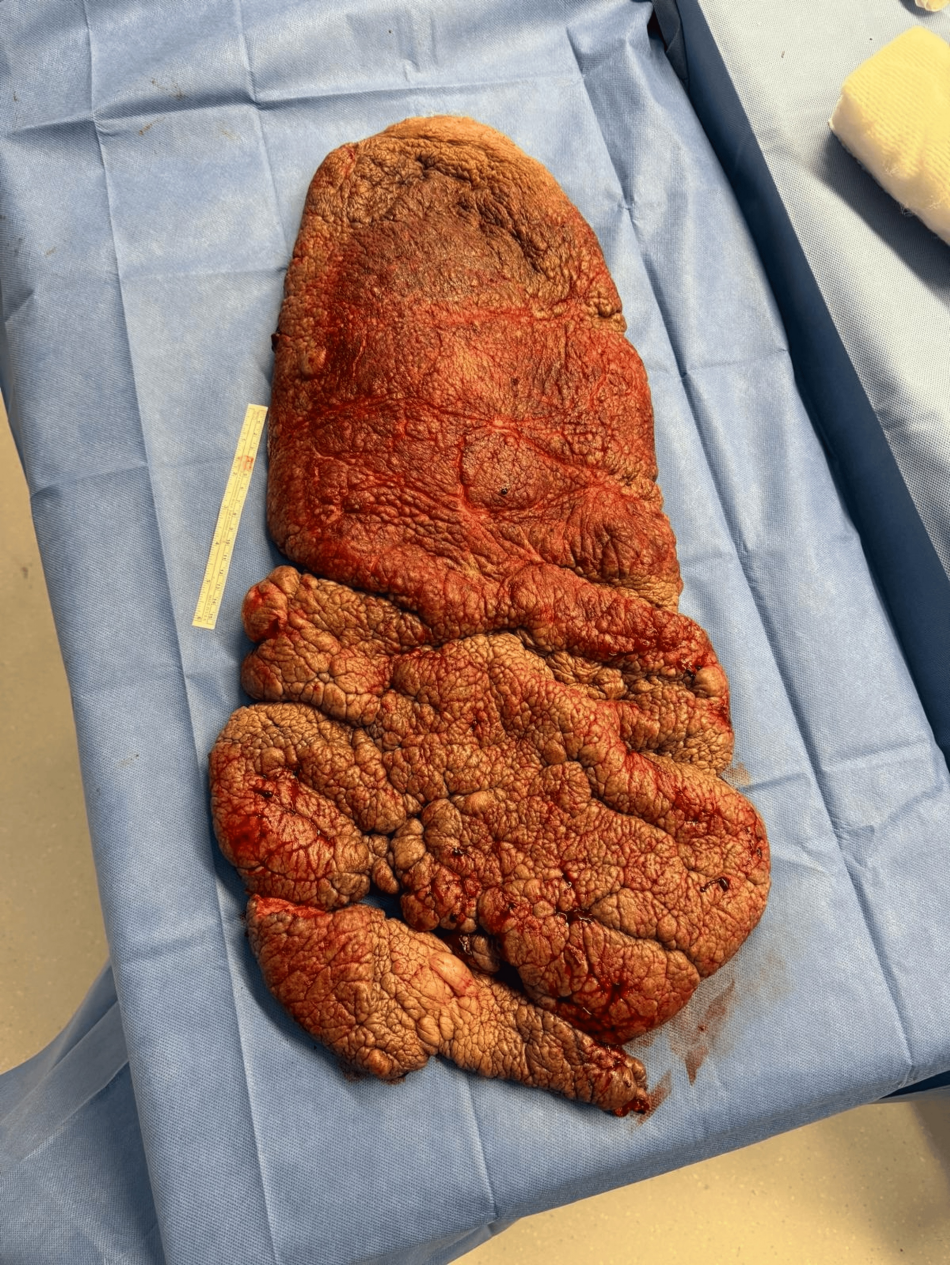
Plexiform neurofibroma after surgical removal, weighing 30 lb and measuring approximately 30 cm × 60 cm.

**Figure 3:**
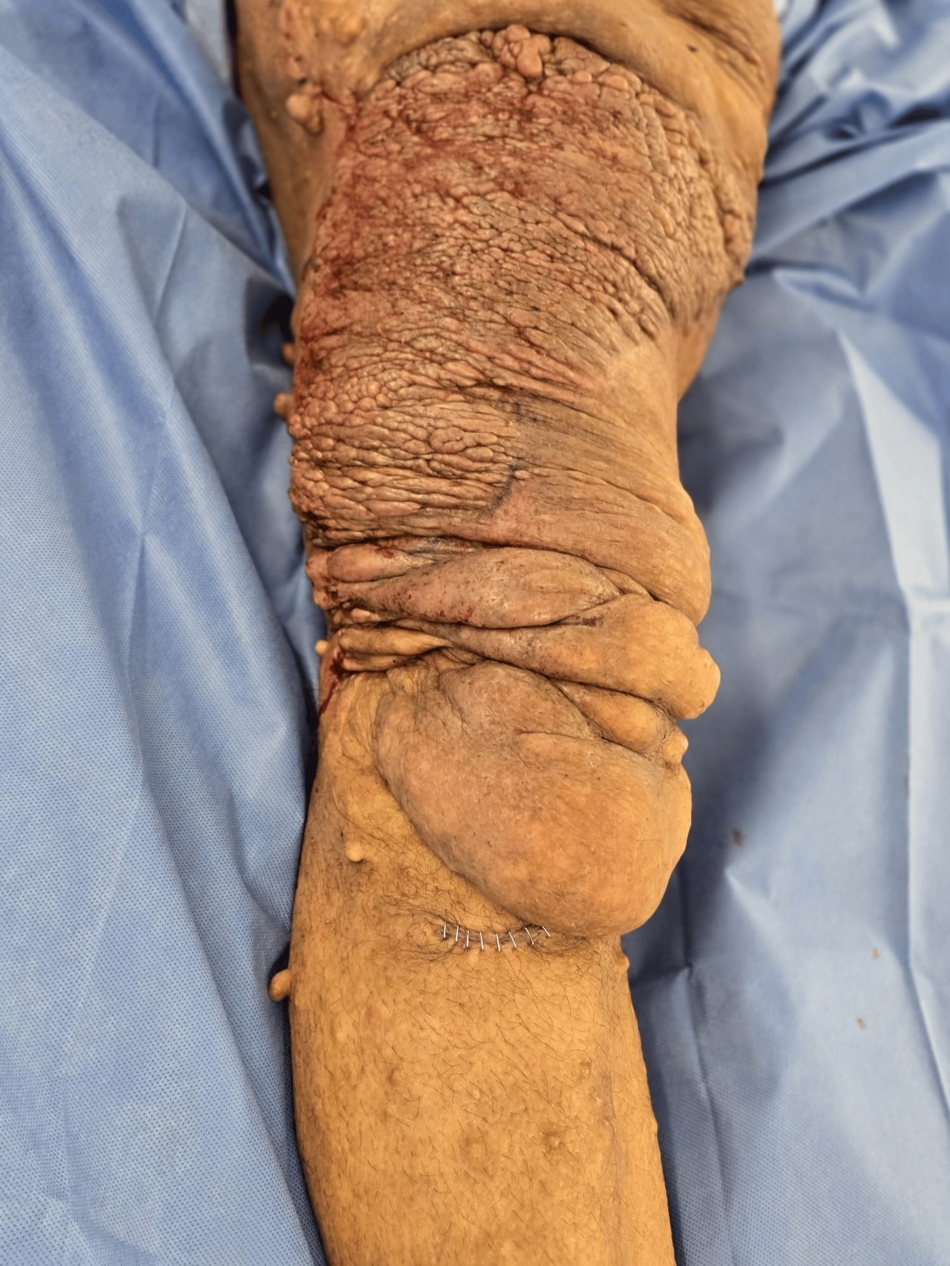
Postoperative appearance of the right lower limb following surgical removal of the plexiform neurofibroma.

Approximately two hours into the procedure, the patient had lost 1,300 ml of the allowable 1,600 ml estimated for the patient. Intraoperative complete blood count (CBC) and arterial blood gases (ABGs) (Table 2) were ordered. Subsequently, two units of packed red blood cells (pRBCs) were initially transfused. Two hours after the first transfusion, another CBC was ordered (Table 3), and an additional unit of pRBCs was transfused. Hemodynamics were maintained throughout the procedure. BP fluctuated between 95/65 mmHg and 135/60 mmHg, HR ranged from 105 to 65 bpm, SpO₂ remained >92%, end-tidal carbon dioxide (etCO₂) ranged from 32 to 45 mmHg, and his temperature was maintained between 35.3 °C and 36.6 °C. Despite blood loss and involuntary movements, the procedure continued, and successful debulking was achieved. A plexiform neurofibroma measuring approximately 60 cm × 30 cm and weighing approximately 30 lb was ultimately removed (Figure 2 and Figure 3).

**Table 2:** First intraoperative CBC and ABGs (2 hours after beginning of procedure). ABG: Arterial blood gas, pCO2: Partial pressure of carbon dioxide, HCO3: Bicarbonate, O2 Sat Calc/meas: oxygen saturation calculated/measured, CO2: carbon dioxide

Test	Results	Normal Range
White Blood Count (X 10^3/uL)	8.1	3.98 - 10.04
Red Blood Count (X 10^6/uL)	3.49	3.93 - 5.22
Hemoglobin (gm/dL)	10.3	11.2 - 15.1
Hematocrit (%)	30.7	36 - 44
Platelets (X 10^3/uL)	224	163 - 369
Standard Base Excess (mEq/L)	-5	−2 - +2
ABG pH (mmHg)	7.39	7.35 – 7.45
ABG pCO2 (mmHg)	34.2	35 - 45
ABG pO2 (mmHg)	218.5	80 - 100
ABG HCO3 (standard) (mEq/L)	21.1	22 - 26
ABG HCO3 (mEq/L)	20	22 - 26
ABG Total CO2 (mEq/L)	21.1	23 - 29
ABG O2 Sat Calc/meas (%)	99.5	95 - 100
ABG Base Excess (mEq/L)	-4.1	−2 - +2

**Table 3:** Second intraoperative CBC (4 hours after beginning of procedure).

Test	Results	Normal Range
White Blood Count (X 10^3/uL)	16.04	3.98 - 10.04
Red Blood Count (X 10^6/uL)	3.97	3.93 - 5.22
Hemoglobin (gm/dL)	11.6	11.2 - 15.1
Hematocrit (%)	34.8	36 - 44
Platelets (X 10^3/uL)	254	163 - 369

The procedure lasted approximately 350 minutes. The patient was extubated after complete recovery by obeying commands. He was transferred to the postoperative anesthesia care unit (PACU) with supplemental oxygen administered via a simple facial mask. Initial PACU vital signs included a BP of 141/100 mmHg, heart rate of 80 bpm, respiratory rate of 14 breaths per minute, and SpO₂ of 98%. He was then transferred to the neurosurgical intensive care unit in stable condition.

On postoperative day six, the patient was evaluated at the bedside by the neurosurgical team. Pain was adequately controlled with acetaminophen and a tramadol/acetaminophen combination regimen. Magnetic resonance imaging (MRI) of the right lower extremity, performed with and without contrast, demonstrated expected postoperative changes without evidence of acute complications. The vacuum-assisted closure device was removed, and the patient continued to exhibit stable motor and vascular function in the right lower extremity. Arrangements were made for ongoing local wound care, and home wound care services were coordinated upon discharge.

## Discussion

The debulking of massive PNs presents significant anesthetic challenges, primarily related to extensive tumor invasion, high vascularity, the risk of major hemorrhage, and the potential for neurophysiologic disturbances during surgical manipulation. These challenges increased in patients with NF1 due to the wide range of potential complications associated with this genetic condition. The combination of PNs and NF1 demands meticulous preoperative planning, heightened intraoperative vigilance, and tailored anesthetic strategies to ensure patient safety and optimize surgical outcomes. In our case, despite appropriate preparation for anticipated challenges, including airway management, hemorrhage risk, and neurophysiological monitoring, the intraoperative course was complicated by unexpected involuntary movements under general anesthesia.

The first anticipated challenge was airway management, which represents a critical anesthetic concern in patients with NF1. Since neurofibromas may involve the tongue, larynx, or trachea, they can lead to airway obstruction and difficult endotracheal intubation [17]. Although our patient experienced a difficult intubation, there was no clinical, radiologic, or anatomic evidence of NF1-related airway complications. Nevertheless, the potential for airway compromise was carefully evaluated and incorporated into perioperative planning.

In addition to airway considerations, the tumor’s marked vascularity and considerable size (62 x 35 cm) raised significant concern for intraoperative hemorrhage. The degree of mass effect and history of prior resections in our patient further heightened concern for hemorrhagic complications, suggesting deep tissue infiltration and a recurrent, aggressive lesion. Moreover, NF1 is associated with underlying vasculopathies that increase the risk of ischemic and hemorrhagic events, underscoring the importance of strict perioperative hemodynamic control [17]. Accordingly, preoperative preparation included cross-matching pRBCs, administration of TXA, and establishment of multiple IV access sites to facilitate rapid fluid and blood product administration. These considerations proved useful in the management of the intraoperative hemorrhage, allowing us to maintain hemodynamic stability throughout the procedure. Our experience aligns with previous data indicating that surgical excision of PNs larger than 13 cm is associated with an increased risk of substantial blood loss and perioperative blood transfusion [18].

The infiltrative nature of the tumor increased the complexity of surgical excision, as multiple neural and adjacent structures were at risk of injury. To mitigate this risk, IONM of the relevant lower extremity muscle groups was employed. A predominantly IV anesthetic technique was selected using propofol, ketamine, lidocaine, and intermittent fentanyl boluses. To preserve the integrity of neurophysiologic signals while maintaining adequate anesthetic depth and hemodynamic stability, propofol and fentanyl were used as they affect IONM to a lesser extent [19-20]. Ketamine was incorporated to enhance IONM signal quality while offsetting propofol-induced hypotension [21], while lidocaine was utilized to reduce overall anesthetic requirements without adversely affecting neurophysiologic monitoring [22]. Low-dose sevoflurane (below 0.5 MAC) was also used for anesthesia maintenance. At this concentration, sevoflurane allows adequate neurophysiological monitoring [23]. No additional neuromuscular blocking agents were used, aside from the succinylcholine administered during induction, as they prevent reliable recording of compound muscle action potentials during MEP monitoring [24].

A unique and clinically significant event in this case was the occurrence of transient, generalized myoclonic jerks. These movements were temporally associated with deep surgical manipulation of the PN adjacent to the femoral artery. During the episode, IONM monitoring demonstrated no abnormalities. Inadequate depth of anesthesia was initially considered, prompting rapid escalation of anesthetic depth with high-dose propofol, ketamine, and opioid infusions. Despite these interventions, the involuntary movements persisted for approximately 30 minutes, suggesting a seizure-like phenomenon (SLP). Although the precise mechanism underlying these movements could not be definitively determined, their unexpected occurrence prompted a systematic evaluation of potential neurologic and anesthetic etiologies.

From a neurological standpoint, individuals with NF1 exhibit a higher prevalence of epilepsy and, consequently, an increased risk of intraoperative seizures compared with the general population [25-26]. Most affected individuals, however, are diagnosed during childhood, with the incidence of new-onset epilepsy decreasing in adulthood [27]. Furthermore, seizures in NF1 are typically associated with underlying intracranial pathology [28]. In the present case, NF1 itself was considered an unlikely etiology for the observed SLP, as the patient had no prior history of epilepsy or evidence of intracranial abnormalities, although this possibility cannot be entirely excluded.

The etiology of the SLP suffered by our patient may be secondary to the use of anesthetic agents. These agents are known to possess both anticonvulsant and proconvulsant properties [29]. Indeed, propofol has been associated with the development of SLP [30]. In patients without epilepsy, SLP most commonly occurs during induction or emergence of anesthesia and typically manifests as generalized tonic-clonic activity [31]. The lower incidence of SLP during the maintenance phase of anesthesia has been attributed, in part, to the concurrent use of neuromuscular blocking agents, which were intentionally avoided in the current case due to IONM requirements. Consequently, propofol may be the underlying cause of the SLP.

Moreover, SLP may have been secondary to fentanyl administration, as opioids have been reported to induce epileptiform activity [29]. Although seizures secondary to opioids are rare, most of the time they represent opioid-induced myoclonus and rigidity rather than true seizures [29]. These involuntary movements may manifest as limb or neck flexion, opisthotonos, or masseter spasm at lower doses, while larger or rapidly administered doses may result in chest wall rigidity and generalized muscle rigidity [32]. Due to these known effects, fentanyl-associated myoclonus was also considered in the differential diagnosis.

Sevoflurane and ketamine may also have epileptogenic properties. Seizure activity associated with sevoflurane has primarily been described at concentrations between 1.5 and 2.0 MAC [33]. In the present case, sevoflurane was maintained at concentrations well below these thresholds, making it an unlikely contributor. While ketamine has demonstrated anticonvulsant effects, it may also induce cortical excitation and seizure-like activity, raising concerns about its potential pro-epileptic effects in susceptible individuals, particularly those with a reduced seizure threshold or underlying neurologic pathology [34]. Moreover, the combination of propofol and ketamine has been shown to exert anticonvulsant rather than proconvulsant effects [29] and has been successfully used for seizure suppression in cases of super-refractory status epilepticus [35]. Therefore, given the concurrent use of propofol and the absence of a known seizure disorder, ketamine was also considered an unlikely primary cause of the observed SLP.

To our knowledge, this is the first reported case of transient generalized myoclonic jerks occurring during debulking surgery of a PN. Although a definitive etiology could not be established, this case underscores the importance of anticipating atypical neuromuscular manifestations during complex NF1-related surgeries, particularly when neuromuscular blockade is limited by IONM. Anesthesiologists should maintain a broad differential diagnosis for intraoperative SLP and recognize that such events may occur despite adequate anesthesia and stable neurophysiologic signals. Heightened vigilance, close multidisciplinary communication, and individualized anesthetic planning remain essential to safely managing the unique perioperative challenges posed by PN resection in patients with NF1.

## Conclusions

This case highlights the anesthetic complexity associated with the surgical debulking of massive plexiform neurofibromas in patients with NF1. In addition to anticipated challenges related to airway management, tumor vascularity, hemorrhage risk, and the need for intraoperative neurophysiological monitoring, the patient developed transient generalized myoclonic jerks temporally associated with deep tumor manipulation in the absence of electrodiagnostic abnormalities. Although the underlying mechanism remains unclear, this finding expands the spectrum of intraoperative neuromuscular phenomena that may appear during PN resection.
